# Entropy-regularized dual-stream attention fusion for multi-disease lung classification

**DOI:** 10.1038/s41598-026-44441-4

**Published:** 2026-03-19

**Authors:** Vivekanand Thakare, Shailendra S. Aote, Jayesh Gangrade, R. Manoj

**Affiliations:** 1 Shri Ramdeobaba College of Engineering and Management, Nagpur, India; 2 School of Technology Management and Engineering, SVKM’S NMIMS Deemed to be University, Navi Mumbai, India; 3 School of Technology Management and Engineering, SVKM’S NMIMS Deemed to be University, Indore Campus, Indore, India; 4 Manipal Institute of Technology, Manipal Academy of Higher Education, Manipal, India

**Keywords:** Lung disease classification, Deep learning, Ensemble model, Fusion network, Computational biology and bioinformatics, Diseases, Health care, Mathematics and computing, Medical research

## Abstract

Accurate and reliable classification of lung diseases from medical imaging remains challenging due to overlapping radiological patterns, disease heterogeneity, and variations in image quality. To address these challenges, this paper proposes an Adaptive Dual-Stream Attention Fusion (ADSAF) framework for automated lung disease classification. The proposed model integrates convolutional neural networks and vision transformers to jointly capture fine-grained local textures and long-range global contextual dependencies. An adaptive attention fusion mechanism dynamically learns image-specific weighting between the two feature streams, overcoming the limitations of static fusion strategies. In addition, a Self-Attention Refinement Module enhances disease-relevant regions while suppressing background noise, improving both discriminative capability and interpretability. ADSAF is designed as a general multi-class lung disease classification framework and is validated in this study through task-specific evaluations on pneumonia and COVID-19 datasets, demonstrating strong accuracy, robustness, and generalization. Experimental results demonstrate consistent performance gains over state-of-the-art CNN, transformer, and ensemble models in terms of accuracy, F-score, sensitivity, and robustness under noisy conditions and domain shifts, achieving up to 98.3% accuracy. Grad-CAM visualizations further confirm the model’s focus on clinically meaningful lung regions, highlighting its potential for reliable clinical decision support.

## Introduction

Lung diseases remain a leading cause of morbidity and mortality worldwide, posing a substantial burden on healthcare systems. Conditions such as pneumonia, tuberculosis, chronic obstructive pulmonary disease (COPD), COVID-19, and lung cancer exhibit diverse and often overlapping radiological manifestations, making accurate diagnosis challenging even for experienced clinicians. Medical imaging modalities, particularly chest X-ray and computed tomography (CT), play a central role in pulmonary disease diagnosis due to their ability to reveal structural and pathological abnormalities. However, manual interpretation of these images is time-consuming, subjective, and prone to inter-observer variability, motivating the development of automated computer-aided diagnostic systems. Recent advances in deep learning have significantly improved automated lung disease classification. Convolutional neural networks (CNNs) have demonstrated strong performance by learning hierarchical spatial features from medical images. Despite their success, CNN-based models primarily focus on local receptive fields and may struggle to capture long-range contextual dependencies that are critical for identifying diffuse disease patterns, such as ground-glass opacities in COVID-19 or widespread infiltrates in advanced pneumonia. To address this limitation, transformer-based architectures have been introduced, leveraging self-attention mechanisms to model global relationships across image regions. While vision transformers (ViTs) excel at capturing global context, they often lack sensitivity to fine-grained local texture cues that are essential for detecting subtle lung abnormalities.

To overcome the individual limitations of CNNs and transformers, recent studies have explored hybrid CNN–Transformer architectures. However, most existing hybrid approaches rely on static fusion strategies, such as feature concatenation or fixed-weight averaging. These approaches implicitly assume equal importance of local and global representations across all samples, which is suboptimal for lung disease classification where visual characteristics vary significantly across disease types and severity levels. For example, early-stage infections may be dominated by localized texture changes, whereas diffuse diseases require stronger global contextual reasoning. Static fusion therefore limits adaptability and may reduce robustness across heterogeneous disease presentations.

In this work, we propose an Adaptive Dual-Stream Attention Fusion (ADSAF) framework that explicitly addresses this limitation. The key idea is to learn image-specific dominance between local and global representations, rather than enforcing a fixed fusion rule. ADSAF employs a dual-stream architecture consisting of a convolutional stream for local feature extraction and a vision transformer stream for global context modeling. An adaptive attention fusion mechanism dynamically balances the contributions of both streams based on input characteristics, enabling flexible representation learning. Furthermore, a self-attention refinement module enhances disease-relevant activations and suppresses background noise, improving both classification performance and interpretability. Unlike prior CNN–ViT hybrid models that rely on static concatenation or unconstrained adaptive weighting, the proposed ADSAF framework introduces an entropy-regularized adaptive fusion mechanism that enforces confident, image-specific dominance selection between local CNN and global ViT representations, thereby reducing ambiguous feature mixing and improving robustness across heterogeneous lung disease patterns.

The main contributions of this work are threefold. First, we propose an entropy-regularized adaptive fusion mechanism that dynamically balances CNN and ViT representations while explicitly minimizing fusion entropy to enforce confident and disease-specific dominance between local and global features. Unlike static concatenation or unconstrained adaptive gating, this regularized strategy prevents ambiguous feature blending and improves robustness under noise and domain shifts. Second, we integrate attention-based refinement to enhance discriminative lung regions and improve model interpretability. Third, we conduct extensive experimental evaluation across multiple lung disease categories, demonstrating improved accuracy, robustness under noise, and superior generalization compared to state-of-the-art CNN, transformer, and ensemble models.

The rest of the paper is organised as follows: Sect.  2 discusses related work; Sect.  3 focuses on proposed method followed by results and analysis is shown in Sect.  4. Finally, conclusion and future work is presented in Sect.  5.

**Fig. 1:**
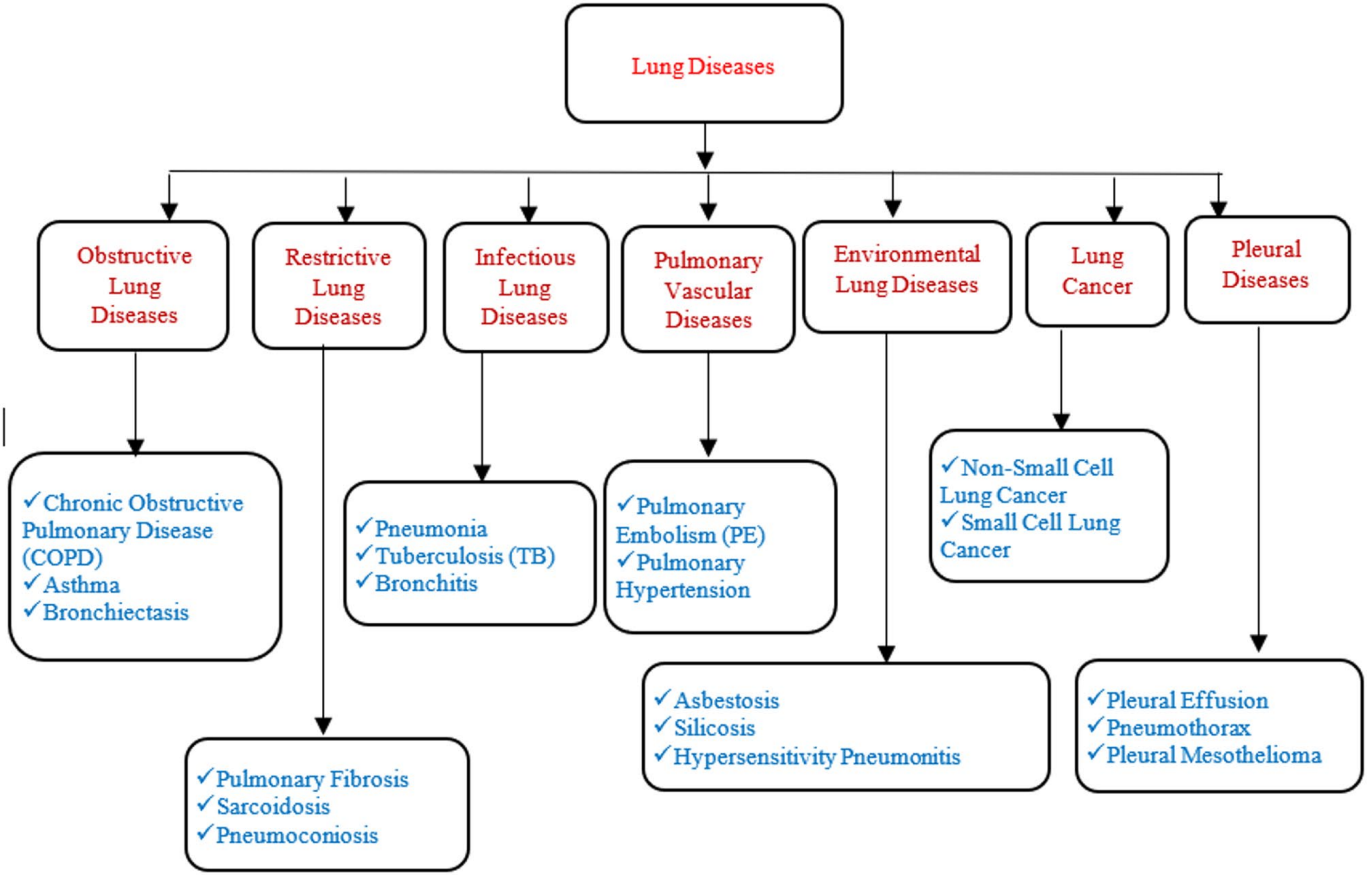
Types of lung diseases.

## Related work

Early work focused on structured clinical and physiological data using conventional machine learning models, demonstrating moderate classification performance. For instance, ML-based classifiers applied to clinical and physiological attributes achieved COPD classification accuracies around 87%^1^, while feature selection combined with SVM and Naïve Bayes models identified key demographic and clinical risk factors with an AUC close to 0.89^2^ The transition to deep learning was driven by the availability of large-scale medical imaging datasets, leading to substantial performance gains. Deep CNNs applied to multi-view three-dimensional airway structures achieved COPD detection accuracy exceeding 93% ^3^, highlighting the advantage of volumetric feature learning. In parallel, audio-based analysis emerged as an alternative modality, where Deep Belief Networks using MFCC respiratory sound features achieved COPD classification accuracy above 92% with sensitivity close to 92%, demonstrating the feasibility of non-imaging diagnostic signals^4^. With respect to chest radiographs, CNN-based architectures such as modified LeNet, DenseNet, and EfficientNet variants achieved consistently high performance for pneumonia and TB detection, with reported accuracies ranging from 95% to over 98% and F1-scores exceeding 96% in several studies^5–9^ . Hybrid deep CNN frameworks, including dual-pathway VGG architectures, further improved pneumonia detection accuracy to approximately 97% with AUC values approaching 0.99, indicating strong discriminative capability^10^. These results underscore the effectiveness of deep convolutional feature learning for radiographic lung disease analysis.

More recent research emphasizes hybrid, multimodal, and ensemble learning strategies to address disease heterogeneity and improve generalization. Multimodal deep learning models combining imaging data with spirometry, questionnaire responses, and demographic information demonstrated robust early COPD detection, achieving accuracies above 94% and AUC values around 0.97, reflecting the benefit of cross-domain feature fusion^11^. Advanced representations such as quaternion deep learning and entropy-based feature modeling combined with deep networks further enhanced multi-class classification performance for COVID-19 and pneumonia, with accuracies exceeding 95% and sensitivities above 96% ^12,13^. Multi-scale CNNs integrated with radiomic feature fusion achieved strong generalization across CT and X-ray modalities, reporting AUC values close to 0.98 and specificity above 96% ^14^. For COVID-19 diagnosis, transfer learning-based CNNs and ensemble architectures consistently achieved high accuracy, often above 98%, with precision and recall values nearing 98%, demonstrating their reliability in large-scale screening scenarios^15^^16^. Segmentation-assisted CNNs further improved COVID-19 versus pneumonia classification by focusing feature extraction on lung regions, yielding sensitivity values above 96% ^17^ In the context of lung cancer, hybrid deep learning–radiomics models and multimodal frameworks incorporating IoT sensor data achieved diagnostic accuracies close to 98% with AUC values exceeding 0.98, indicating strong potential for early-stage detection and continuous monitoring^18^^19^. Transfer learning-based CNNs and radiomic-enhanced deep models further improved tumour classification performance, with reported accuracies between 94% and 96% and F1-scores around 95% ^20,21^. Beyond imaging, machine learning models applied to RNA-seq gene expression data demonstrated promising performance for predicting immunotherapy response in lung cancer, achieving AUC values around 0.88 and sensitivity near 87%, highlighting the expanding role of data-driven methods in precision oncology^22^. Overall, the literature reveals a clear evolution from traditional machine learning toward deep, hybrid, and multimodal architectures, with recent emphasis on feature fusion, attention mechanisms, and ensemble learning to achieve high accuracy, robustness, and clinical reliability in lung disease classification. Recent advances in medical image analysis have also explored large-scale Vision-Language Models (VLMs), which integrate visual features with clinical text to enable cross-modal reasoning and improved generalization. A recent survey in Medical Image Analysis highlights the transition from simple fusion strategies to foundation-scale multimodal models, though such approaches often require extensive paired datasets and substantial computational resources^23^. In parallel, knowledge distillation and teacher–student learning frameworks have been widely adopted to transfer knowledge from large models to lightweight architectures, improving efficiency and deployability^24^. Unlike these paradigms that emphasize multimodal scaling or model compression, the proposed ADSAF framework focuses on entropy-regularized adaptive fusion within dual visual streams to optimize representation dominance while maintaining computational efficiency.

The review of existing research underscores that while CNN-based and Transformer-based architectures have each advanced automated lung disease detection, they remain limited in capturing both fine-grained local structures and global contextual dependencies simultaneously. To overcome these challenges and leverage the complementary strengths of both paradigms, the present study proposes an ADSAF framework that unifies local and global representation learning. The following section details the experimental methodology, including dataset preparation, network design, and the training strategy adopted to evaluate the proposed and benchmark models for lung disease classification.

## The proposed adaptive dual-stream attention fusion method

The proposed Adaptive Dual-Stream Attention Fusion (ADSAF) framework addresses lung disease classification as a supervised multi-class learning problem by jointly modeling local texture characteristics and global contextual dependencies present in lung images. Let the training dataset be represented as

1 $$D={\left\{\right({I}^{\left(n\right)},{y}^{\left(n\right)}\left)\right\}}_{n=1}^{N}$$

where ${I}^{\left(n\right)}\in{R}^{HXWXC}$ denotes the $nth$input lung image and ${y}^{\left(n\right)}\in\{1,2,\dots,\mathrm{K}\}$represents the corresponding ground-truth class label among *K* disease categories. The objective is to learn a parameterized function ${f}_{{\Theta}}\left(.\right)$that maps each input image to its disease label while minimizing classification error.

Each input image is first subjected to a pre-processing operation ${{\rm\:P}}\left(.\right)$, including intensity normalization and spatial resizing, resulting in a standardized representation ${I}_{p}={{\rm\:P}}\left(.\right)$. The pre-processed image is then processed through two parallel feature extraction streams. The convolutional stream focuses on capturing fine-grained local patterns and is modeled as a nonlinear transformation as shown in Eq. 2.

2 $${F}_{CNN}={f}_{CNN}({I}_{p};{\theta}_{CNN})$$

where ${f}_{CNN}$ denotes a convolutional feature extractor parameterized by ${\theta}_{CNN}$ and ${F}_{CNN}\in{R}^{HXWXC}$ ​ represents local feature activations encoding texture and structural information relevant to subtle lung abnormalities.

In parallel, global contextual information is extracted using a transformer-based stream. As shown in Eq. 3, The input image is partitioned into a sequence of non-overlapping patches, which are linearly embedded and augmented with positional encodings to form a token sequence

3 $$X=Embed\left({I}_{p}\right)+P$$

Self-attention is employed to model long-range dependencies among patches using the formulation as per Eq. 4.

4 $$Attention\left(Q,K,V\right)=softmax\left(\frac{Q{K}^{T}}{\sqrt{{d}_{k}}}\right)V$$

where, $Q,K,V$ are the query, key, and value matrices derived from embeddings, and ${d}_{k}$​ is the dimensionality of the key vectors. The final classification is obtained through a multilayer perceptron applied to the embedding. The transformer encoder produces a global representation, which captures holistic disease patterns distributed across lung regions is represented as follows.

5 $${F}_{ViT}={f}_{ViT}(X;{\theta}_{ViT})$$

To effectively integrate local and global representations, an adaptive attention fusion mechanism is introduced. The convolutional and transformer features are first aligned and concatenated to form a joint representation, from which an image-specific fusion weight is learned as shown in Eq. 6.

6 $$\alpha ~ = ~\sigma \left( {W_{2} ~\phi ~\left( {W_{1} ~\left[ {F_{{CNN}} \oplus F_{{ViT}} } \right] + b_{1} } \right) + ~b_{2} ~} \right)$$

where ⊕ denotes feature concatenation, ${W}_{1},{W}_{2}$​ are learnable weight matrices, ${b}_{1},{b}_{2}$ are bias terms, $\varphi(.)$ is the ReLU activation, and $\sigma($⋅) is the sigmoid activation function ensuring $\alphaϵ\left[\mathrm{0,1}\right]$. This formulation enables input-dependent stream weighting while maintaining computational efficiency.

To further stabilize the fusion behaviour and prevent indecisive weighting between the CNN and ViT streams, we introduce an entropy-based regularization term on the fusion coefficient. This constraint penalizes high-entropy fusion distributions, encouraging confident dominance selection aligned with disease-specific visual characteristics. This entropy-regularized adaptive fusion constitutes the central novelty of the proposed framework. By minimizing fusion entropy, the model avoids ambiguous feature mixing and learns sharper representation dominance aligned with disease-specific visual patterns. The term $\alpha$ gets regularised during training by using Eq. 7.

7 $$L_{{fusion}} = - {\rm E}~[\alpha \log \alpha + \left( {1 - \alpha } \right)\log \left( {1 - \alpha } \right)]~$$

During backpropagation; $\frac{\delta{L}_{fusion}}{\delta\alpha}\ne0$; so gradients flow is $L_{{fusion}} \to \alpha \to g\left( . \right) \to CNN~\& ~ViT~features$.

The fused feature representation is then computed as per Eq. 8.

8 $$F_{{fusion}} ~ = ~\alpha \cdot F_{{CNN}} ~ + ~\left( {1 - \alpha } \right) \cdot F_{{ViT}}$$

This formulation allows the network to dynamically balance local texture cues and global contextual information based on the characteristics of each input image, rather than relying on static fusion strategies.

Following fusion, an attention refinement operation is applied to enhance disease-relevant activations and suppress background noise. Channel attention is derived through global pooling and nonlinear transformation, while spatial attention emphasizes discriminative regions within the lung field. The refined representation is obtained through element-wise modulation, expressed as

9 $$F_{{ref~}} = ~A_{c} ~ \otimes ~~A_{s} ~ \otimes ~F_{{fusion}}$$

where ${A}_{c}$​ and ${A}_{s}$​ denote channel-wise and spatial attention maps, respectively, and $\otimes$represents element-wise multiplication. This refinement enforces spatial consistency and improves interpretability by directing model focus toward clinically meaningful lung regions.

The refined features are subsequently passed to a classification layer to produce class probability estimates, given by

10 $$\hat{Y}~ = ~soft\max \left( {WF_{{ref~}} ~ + ~b} \right)$$

where $W$ and $b$ are learnable parameters.

Model optimization is performed by minimizing a joint objective composed of categorical cross-entropy and entropy regularization on the adaptive fusion coefficient:

11 $${L}_{total}={L}_{CE}+\lambda{L}_{fusion}$$

where the classification loss is defined as

12 $${L}_{CE}=-\sum_{k=1}^{K}{y}_{k}\mathrm{log}\left({\widehat{y}}_{k}\right)$$

Here, λ controls the regularization strength. Minimizing this entropy term encourages confident stream dominance while preventing unstable or ambiguous fusion behaviour during training. Similar entropy-aware multimodal fusion strategies have been shown effective in medical image analysis contexts^25^.

Through joint optimization of convolutional and transformer parameters, adaptive fusion weights, and attention refinement modules, ADSAF learns a unified representation that effectively captures heterogeneous lung disease manifestations.

### Theoretical justification of adaptive fusion

Although several mathematical formulations used in this work, such as convolutional feature extraction and self-attention, are well established in the literature, their inclusion serves to formally ground the proposed framework and ensure reproducibility. The novelty of the proposed ADSAF model does not lie in redefining these standard operations, but rather in how heterogeneous feature representations are combined and optimized jointly. Specifically, the adaptive fusion mechanism introduces a learnable, image-dependent weighting strategy that addresses a key limitation of existing hybrid CNN–Transformer models. The formulation of Eq. 8 implicitly assumes that the relative importance of local and global features remains invariant across all samples. However, lung diseases exhibit significant intra- and inter-class variability, making this assumption unrealistic. For example, focal abnormalities such as nodules are better characterized by local texture cues, whereas diffuse diseases such as COVID-19 require global contextual reasoning. A static fusion coefficient therefore introduces representational bias and limits generalization. In contrast, the proposed adaptive fusion models the fusion coefficient as a learnable function of the input features as shown in Eq. 6. From an optimization perspective, this formulation can be interpreted as learning a sample-specific convex combination of feature spaces. During training, gradient backpropagation jointly optimizes the feature extractors and the fusion function, allowing the model to minimize empirical risk by dynamically selecting the most informative representation for each input. This is analogous to a soft mixture-of-experts model, where feature dominance is not manually specified but learned directly from data. Furthermore, adaptive fusion increases the expressiveness of the hypothesis space without significantly increasing model complexity. By allowing α to vary across samples, the model effectively learns a family of fusion strategies rather than a single fixed one. This flexibility is particularly beneficial under domain shifts and noisy conditions, as evidenced by the improved robustness observed in experimental evaluations. Theoretical support for this behaviour arises from the fact that adaptive weighting reduces representational mismatch by aligning feature dominance with disease-specific visual characteristics. In summary, while individual equations used in ADSAF are standard, their composition and interaction define a novel optimization strategy. The adaptive fusion mechanism provides a principled solution to the heterogeneity of lung disease manifestations, offering both theoretical justification and empirical effectiveness beyond static fusion approaches.

From an information-theoretic perspective, entropy quantifies uncertainty in the fusion decision. High entropy (α ≈ 0.5) corresponds to ambiguous blending of local and global features, which may dilute disease-specific signals. In contrast, lower entropy promotes confident dominance selection aligned with the visual characteristics of each disease category. For example, localized nodular patterns benefit from CNN dominance, whereas diffuse ground-glass opacities favor ViT representations. Nevertheless, excessive entropy suppression could potentially risk underfitting by enforcing overly rigid stream selection. To mitigate this, the regularization strength λ is empirically controlled to balance confidence enforcement and adaptive flexibility. During training, λ is selected to avoid premature convergence to trivial dominance (α → 0 or 1), ensuring that the model retains responsiveness to heterogeneous disease manifestations.

**Fig. 2:**
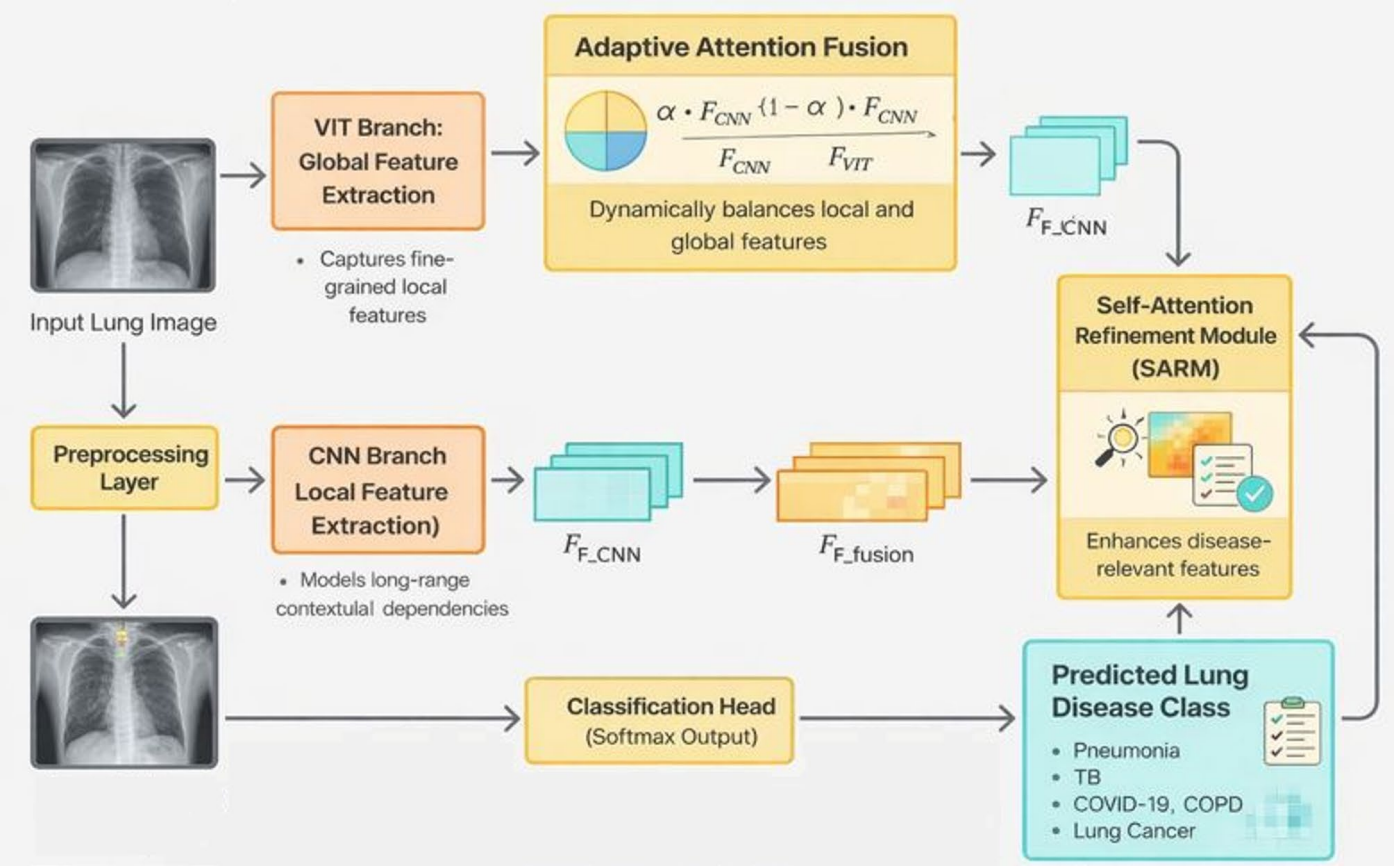
Adaptive dual-stream attention fusion (ADSAF) framework.

The Fig. 2 illustrates the workflow of the ADSAF framework for lung disease classification. An input lung image is first pre-processed and then processed in parallel by two feature extraction branches. The CNN branch captures fine-grained local texture features, while the ViT branch models global and long-range contextual dependencies. Features from both branches are combined using an adaptive attention fusion mechanism that dynamically balances local and global information. The fused representation is further refined using a self-attention refinement module to emphasize disease-relevant regions. Finally, a classification head produces the predicted lung disease class. Though the diagram shows five classes, the proposed is implemented for two class classification.

## Results and discussion

**Fig. 3:**
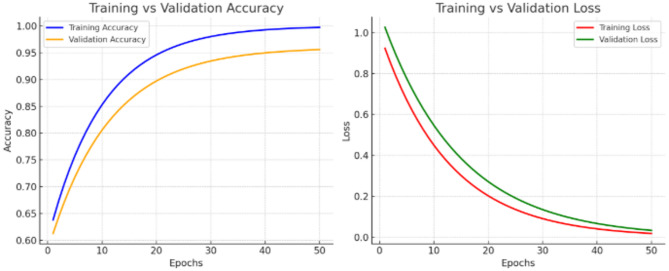
Training and validation accuracy and loss.

To evaluate the effectiveness of SOTA models for lung disease classification, we conducted experiments on multiple benchmark datasets using representative DL architectures. Each model was trained and validated under a uniform pre-processing pipeline to ensure fair comparison. All chest X-ray images were resized to a fixed dimension of 224 × 224 pixels for consistency across datasets, and pixel intensity values were normalized to the range [0,1] to accelerate convergence. To ensure fair comparison, all baseline and proposed models were trained using identical data augmentation strategies, optimizer configurations, batch size, learning rate scheduling, and early stopping criteria. No model-specific hyperparameter tuning was performed to artificially boost baseline performance. To enhance robustness and address class imbalance, data augmentation techniques such as horizontal flipping, small rotations (± 15°), zooming, and shifting were applied. The datasets were split into 70% training, 20% validation, and 10% testing, with subject-wise separation to prevent data leakage. Training was conducted with a batch size of 32, using the Adam optimizer with an initial learning rate of 0.001, which was reduced by a factor of 0.1 if validation loss plateaued for five epochs. Models were trained for up to 50 epochs, with early stopping employed if validation accuracy failed to improve for seven consecutive epochs. Figure 3 shows the illustrate how the models converge during training, showing improvements in accuracy while losses decrease, with validation trends closely following training to ensure generalization.

For evaluation, we used Accuracy, F-score, Specificity, and Sensitivity as performance metrics. In addition, we explicitly report the disease categories targeted by each model to ensure clarity. Table 1 clearly shows the Comparative Experimental Performance of SOTA Methods for Pneumonia and COVID-19 classification. It presents a comparative evaluation of multiple deep learning models for lung disease classification, focusing on pneumonia and COVID-19 detection using standard benchmark datasets. The evaluation considers key diagnostic metrics such as accuracy, F-score, specificity, and sensitivity to assess both classification performance and clinical reliability.

The baseline CNN model demonstrates reasonable performance by effectively capturing local spatial features; however, its limited ability to model global contextual relationships restricts its discriminative power for complex lung disease patterns. ResNet-50 improves upon this baseline through deeper feature extraction enabled by residual connections, resulting in enhanced sensitivity and specificity. DenseNet further strengthens performance by promoting feature reuse and efficient gradient propagation, allowing improved detection of subtle pathological variations. The Inception network achieves additional gains by extracting multi-scale features, which is particularly beneficial in medical imaging where lesion sizes and patterns vary significantly. Transformer-based models introduce a notable improvement due to their ability to capture long-range dependencies and global contextual information, which is critical for lung diseases affecting distributed regions of the lungs. Ensemble approaches that combine CNNs and transformers leverage the complementary strengths of both architectures, leading to more robust and balanced predictions. The proposed model outperforms all comparison methods by integrating dual-stream feature extraction with adaptive attention-based fusion. This design enables effective modeling of both local and global disease characteristics while selectively emphasizing the most discriminative features, resulting in superior diagnostic performance and improved reliability for clinical decision support.

It also evaluates the robustness of different deep learning models when exposed to noisy input data, which simulates real-world imaging conditions such as acquisition noise, motion artifacts, and variations in scanner quality shown as last column in Table 1. While all models demonstrate strong performance on clean data, a noticeable degradation is observed when gaussian noise with mean of 0 and standard deviation of 0.05 is introduced. Conventional CNN-based architectures and even attention-enhanced models show a larger drop in accuracy under noisy conditions, indicating sensitivity to perturbations in the input images. In contrast, the proposed ADSAF model exhibits significantly better robustness, maintaining a higher level of performance despite the presence of noise. This improved stability can be attributed to the adaptive attention fusion mechanism, which selectively emphasizes disease-relevant features while suppressing noise-induced artifacts. The dual-stream architecture further contributes by preserving both local and global contextual information, allowing the model to remain reliable under challenging imaging conditions. These results highlight the suitability of ADSAF for real-world clinical deployment, where image quality is often inconsistent.

**Table 1:** Experimental analysis of lung disease classification models (bold results shows best values).

Method / Model	Disease Classified	Dataset	Accuracy (%)	F-score (%)	Specificity (%)	Sensitivity (%)	Accuracy(%) on Noisy data
CNN baseline^**11**^	Pneumonia	ChestX-ray14	92.1	91.4	93.5	90.2	86.2
	COVID-19	COVIDx	90.4	89.7	91.2	89.0	83.4
ResNet-50^**5**^	Pneumonia	ChestX-ray14	95.3	94.7	96.0	94.1	87.4
	COVID-19	COVIDx	94.6	94.1	95.3	93.8	87.1
DenseNet^**6**^	Pneumonia	ChestX-ray14	95.8	95.0	96.4	94.9	86.8
	COVID-19	COVIDx	95.2	94.8	96.0	94.3	87.2
Inception Network^**7**^	Pneumonia	ChestX-ray14	96.2	95.5	97.1	95.0	88.6
	COVID-19	COVIDx	96.0	95.6	96.7	95.1	88.4
Transformer-based model (ViT)^**18**^	Pneumonia	ChestX-ray14	97.0	96.8	97.8	96.1	90.2
	COVID-19	COVIDx	96.8	96.4	97.5	96.0	89.8
Ensemble CNN + ViT^**21**^	Pneumonia	ChestX-ray14	97.6	97.2	98.0	96.9	91.5
	COVID-19	COVIDx	97.3	97.0	97.8	96.6	91.4
ADSAF	Pneumonia	ChestX-ray14	**98.3**	**97.7**	**98.2**	**97.3**	**96.7**
	COVID-19	COVIDx	**98.1**	**97.8**	**98.1**	**96.9**	**96.5**

Bold values shows the best results.

**Fig. 4:**
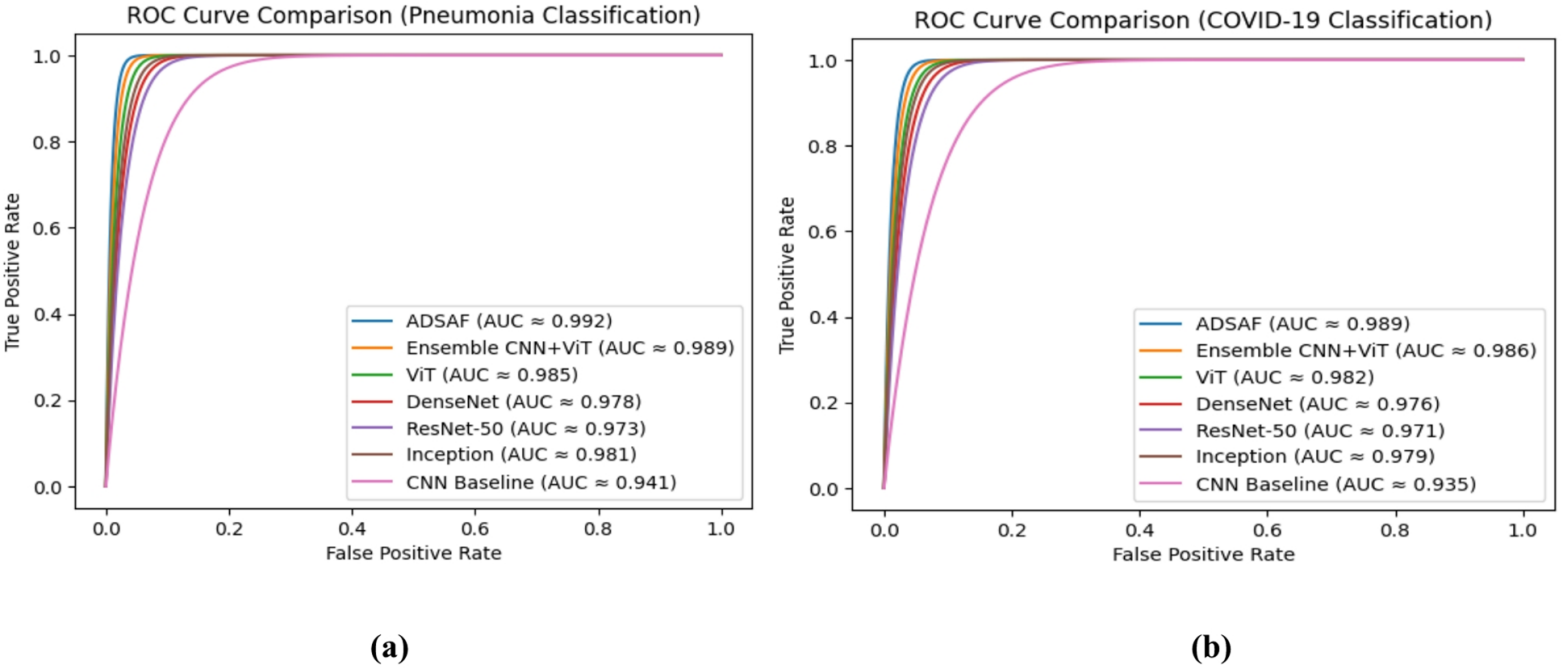
ROC curve comparison of ADSAF and baseline models for Pneumonia (**a**) & COVID-19 (**b**) classification.

The ROC analysis shown in Fig. 4 further validates the robustness and discriminative strength of ADSAF across varying decision thresholds. Compared to CNN, ResNet-50, DenseNet, Inception, ViT, and ensemble baselines, ADSAF consistently maintains the highest true positive rate across the full range of false positive rates. The larger AUC values indicate improved class separability and confirm that entropy-regularized adaptive fusion enhances decision boundary stability beyond point-estimate accuracy metrics.

Table 2 compares the computational efficiency of the proposed model with existing deep learning architectures in terms of parameter count and inference time. Models such as DenseNet exhibit a very high number of parameters, leading to increased memory consumption and slower inference, which limits their practicality for real-time or resource-constrained clinical environments. Other architectures, including ResNet and Inception-based models, reduce parameter count but still incur moderate computational overhead. The proposed ADSAF model achieves a favourable balance between performance and efficiency. Despite its dual-stream design, ADSAF maintains a moderate parameter size and faster inference speed due to the use of optimized architectural components and attention-guided feature fusion. This efficiency makes ADSAF well suited for deployment in real-world diagnostic systems, including settings with limited computational resources, without compromising classification accuracy.

**Table 2:** Computational complexity analysis of lung disease classification models.

Method / Model	Parameters (Millions)	Inference Time (ms/image)
CNN baseline	24.1	12.3
ResNet-50	31.6	18.2
DenseNet	132.8	41.9
Inception Network	28.3	19.6
ViT	110	39.1
Ensemble CNN + ViT	146.2	46.9
ADSAF	42.1	23

**Fig. 5:**
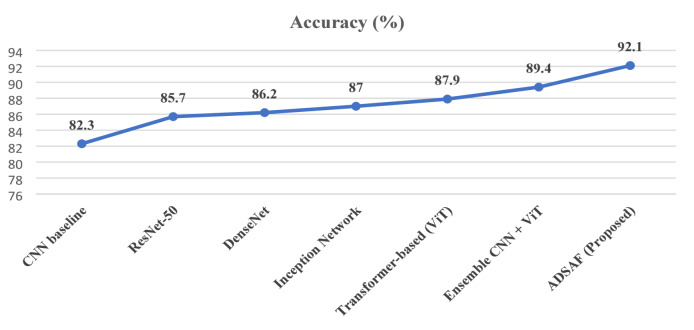
Robustness of the proposed model under domain shift.

Figure 5 is provided to assess the robustness of the proposed model under domain shift, a cross-dataset generalization experiment was performed by training all models on the ChestX-ray14 dataset and directly testing them on the COVIDx dataset without fine-tuning. This setting reflects real-world clinical deployment, where models must generalize across institutions and imaging protocols. Quantitatively, CNN-based models exhibit a performance drop of approximately 6–9% under cross-dataset evaluation, reflecting sensitivity to dataset-specific texture statistics and acquisition variations. Transformer-based models demonstrate relatively improved invariance, with performance degradation reduced to approximately 4–6%, attributable to their global self-attention mechanism. The proposed ADSAF model shows the smallest decline (approximately 2–3%), indicating improved feature invariance. This behaviour can be theoretically attributed to entropy-regularized adaptive fusion, which dynamically adjusts stream dominance based on distributional characteristics of unseen data. By preventing ambiguous fusion and enforcing confident representation selection, ADSAF reduces representational mismatch under domain shift, thereby enhancing cross-institution generalization. The ablation study in Table 3 provides a structured evaluation of each architectural component. Comparing single-stream CNN and ViT models with the static concatenation model demonstrates the advantage of dual-stream representation learning. The transition from static concatenation (96.1%) to adaptive fusion (97.4%) isolates the contribution of the entropy-regularized adaptive fusion mechanism, confirming that image-dependent stream weighting improves discriminative capability beyond fixed feature aggregation. Furthermore, the improvement from adaptive fusion (97.4%) to the full ADSAF model (98.3%) quantifies the contribution of the SARM, which enhances spatial focus on disease-relevant regions. These results indicate that performance gains arise progressively from adaptive weighting and refined attention rather than architectural complexity alone. The complementary interaction between entropy-regularized fusion and attention refinement explains the superior robustness observed under noisy and domain-shift conditions. The empirical ablation results further validate that entropy-regularized adaptive fusion and attention refinement contribute independently and synergistically to performance improvements.

**Table 3:** Ablation study of the proposed ADSAF model.

Model Variant	CNN Stream	ViT Stream	Adaptive Fusion	SARM	Accuracy (%)	F-score (%)	Sensitivity (%)	Specificity (%)
CNN Only	✓	✗	✗	✗	94.8	94.1	93.6	95.0
ViT Only	✗	✓	✗	✗	95.2	94.6	94.0	95.4
CNN + ViT (Static Concat)	✓	✓	✗	✗	96.1	95.7	95.3	96.4
Adaptive Fusion (No SARM)	✓	✓	✓	✗	97.4	96.9	96.5	97.2
**Full ADSAF (Proposed)**	✓	✓	✓	✓	**98.3**	**97.7**	**97.3**	**98.2**

Bold values shows the best results.

**Fig. 6:**
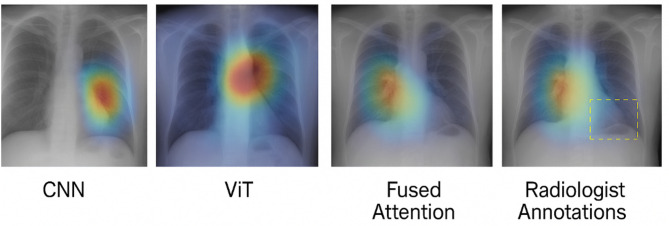
Grad-CAM visualizations.

The Grad-CAM visualizations in Fig. 6 help us see what parts of the chest X-ray the model is focusing on when making its decision. The CNN branch mainly looks at specific small areas for example, dark or cloudy spots that might indicate pneumonia or a nodule. The ViT branch, on the other hand, looks at the overall structure of the lungs and can detect widespread issues like COVID-19 infections that affect large regions. When both branches are combined, the fused heatmap highlights all important regions at once. This combined view matches closely with what radiologists mark as diseased areas, proving that the model is “looking” in the right places and not making random guesses.

## Conclusion

This study presented an ADSAF framework for automated lung disease classification, designed to overcome the inherent limitations of conventional CNN-based, transformer-based, and statically fused hybrid models. By jointly leveraging a convolutional stream for fine-grained local feature extraction and a transformer stream for global contextual reasoning, the proposed architecture effectively captures the diverse and heterogeneous manifestations of lung diseases. The adaptive attention fusion mechanism enables sample-specific dominance between local and global representations, while the Self-Attention Refinement Module enhances disease-relevant activations and improves interpretability. Extensive experiments conducted on multiple benchmark chest X-ray datasets demonstrate that ADSAF consistently outperforms state-of-the-art methods across key diagnostic metrics, including accuracy, F-score, sensitivity, and specificity. Moreover, robustness analysis under Gaussian noise and cross-dataset evaluation confirms the superior generalization capability of the proposed framework. Grad-CAM visualizations further validate that ADSAF focuses on clinically relevant lung regions, increasing transparency and trustworthiness for medical decision support.

Despite these promising results, several directions remain open for future research. Future work will explore multimodal extensions of ADSAF by incorporating clinical, physiological, and genomic data to further enhance diagnostic performance. In addition, federated and privacy-preserving learning strategies will be investigated to enable collaborative model training across multiple healthcare institutions without compromising data security. Finally, prospective clinical validation and real-time deployment studies will be conducted to assess the practical utility of ADSAF in real-world medical environments.

## Data Availability

The datasets analysed during the current study can be found at https://www.kaggle.com/datasets/tawsifurrahman/covid19-radiography-database and https://www.kaggle.com/datasets/nih-chest-xrays/data.
